# Navigating the unexplored seascape of pre-miRNA candidates in single-genome approaches

**DOI:** 10.1093/bioinformatics/bts574

**Published:** 2012-10-10

**Authors:** Nuno D. Mendes, Steffen Heyne, Ana T. Freitas, Marie-France Sagot, Rolf Backofen

**Affiliations:** ^1^Équipe BAOBAB, Laboratoire de Biométrie et Biologie Évolutive (UMR 5558), CNRS, University of Lyon 1, Lyon 69622, Villeurbanne Cedex, France, ^2^INESC-ID/IST, 9 Rua Alves Redol, 1000-029 Lisbon, Portugal, ^3^BAMBOO Team, INRIA Rhône-Alpes, 655 Avenue de l’Europe, 38330 Montbonnot Saint-Martin, France, ^4^Instituto Gulbenkian de Ciência, Rua da Quinta Grande 6, 2780-156 Oeiras, Portugal, ^5^Department of Computer Science, ^6^Center for Biological Signalling Studies (BIOSS), University of Freiburg, 79110 Freiburg, Germany and ^7^Center for Non-coding RNA in Technology and Health, University of Copenhagen, Grønnegårdsvej 3, 1879 Frederiksberg C, Denmark

## Abstract

**Motivation:** The computational search for novel microRNA (miRNA) precursors often involves some sort of structural analysis with the aim of identifying which type of structures are prone to being recognized and processed by the cellular miRNA-maturation machinery. A natural way to tackle this problem is to perform clustering over the candidate structures along with known miRNA precursor structures. Mixed clusters allow then the identification of candidates that are similar to known precursors. Given the large number of pre-miRNA candidates that can be identified in single-genome approaches, even after applying several filters for precursor robustness and stability, a conventional structural clustering approach is unfeasible.

**Results:** We propose a method to represent candidate structures in a feature space, which summarizes key sequence/structure characteristics of each candidate. We demonstrate that proximity in this feature space is related to sequence/structure similarity, and we select candidates that have a high similarity to known precursors. Additional filtering steps are then applied to further reduce the number of candidates to those with greater transcriptional potential. Our method is compared with another single-genome method (TripletSVM) in two datasets, showing better performance in one and comparable performance in the other, for larger training sets. Additionally, we show that our approach allows for a better interpretation of the results.

**Availability and Implementation:** The MinDist method is implemented using Perl scripts and is freely available at http://www.cravela.org/?mindist=1.

**Contact:** backofen@informatik.uni-freiburg.de

**Supplementary information:** Supplementary data are available at *Bioinformatics* online.

## 1 INTRODUCTION

MicroRNAs (miRNAs) constitute one of several classes of small RNAs found in plant and animal branches of Eukaryota. Since the discovery of the first miRNAs in *Caenorhabditis elegans* (Lee *et al.*, 1993), an abundant number of these regulatory RNAs (ranging from 18 to 25 nt in length) have been discovered and their underlying mechanisms investigated [for an overview see e.g. Bartel (2009)]. MiRNAs originate from the maturation of larger precursors of approximately 70 nt called pre-miRNAs.

An important feature of pre-miRNAs that elicits their recognition by the miRNA-processing machinery is their secondary structure. Pre-miRNAs typically exhibit a stem-loop structure with few internal loops or asymmetric bulges but the variety of structures that are efficiently recognized has escaped any strict characterization (Lindow and Gorodkin, 2007). Previously, we proposed a combination of measures that distinguishes true pre-miRNAs from the large number of stem-loops that can be found in metazoan genomes (Mendes *et al.*, 2010). However, the number of precursor candidates (in the order of a few hundred thousand) obtained above the optimal cutoff of the score, which combines measures of stability and robustness, is still impractically large to be subjected to experimental confirmation (see the CRAVELA framework website for further details: http:///www.cravela.org). Despite the fact that all these candidates consist of or contain stem-loops, the details of their secondary structure have not been subjected to a thorough analysis.

The most immediate approach to analyzing the variety of pre-miRNAs in our candidate set is to seek the identification of structural families amongst the precursor candidates. Although miRNAs have been grouped into families according to their sequence similarity in the miRBase database (Griffiths-Jones *et al.*, 2003), this approach does not give enough insight as to the structural features that are important for the recognition by the miRNA-processing machinery. Hence, the grouping has to be performed according to sequence and structure. Various algorithmic approaches have been introduced to determine structural similarities and to derive consensus structure patterns for structural RNAs with low sequence identity (Bompfunewerer *et al.*, 2008; Bradley *et al.*, 2008; Gorodkin *et al.*, 1997; Havgaard *et al.*, 2005; Heyne *et al.*, 2009; Höchsmann *et al.*, 2003; Hofacker *et al.*, 2004; Mathews and Turner, 2002; Sankoff, 1985; Siebert and Backofen, 2005; Will *et al.*, 2007). A first approach toward the clustering of miRNAs has been achieved in Kaczkowski *et al.* (2009), where a sequence–structure alignment was used to cluster 220 miRNAs into structural classes. However, all these approaches suffer from a high computational complexity, with a time requirement typically between *O*(*n*^4^) and *O*(*n*^6^). It is thus computationally unfeasible to cluster hundreds of thousands of candidates using this approach.

Thus, instead of trying to cluster the candidate set, we summarize the structural and sequence features of each candidate using a vectorial representation and attempt to identify the region of the feature space most likely to contain hairpins recognized by the cellular miRNA-processing machinery. Furthermore, using samples of the candidate set, we show that the relative positions of the representations of the candidates in the feature space are reminiscent of the partitions derived from a conventional clustering performed with the state-of-the-art sequence/structure alignment tool LocARNA (Will *et al.*, 2007). And, most importantly, we observe that known precursors are represented in a limited portion of the feature space.

We use this approach to analyse a set of robust and stable hairpins extracted from a genome-wide scan (Mendes *et al.*, 2010) of *Anopheles gambiae* and *Drosophila melanogaster*, greatly reducing the number of candidates. A further reduction is achieved by assessing the transcriptional potential of each remaining candidate and, by additionally restricting our analysis to candidates with the potential of being part of miRNA genomic clusters, we obtain a dataset which is small enough to be subjected to experimental verification.

## 2 MATERIALS AND METHODS

In this work, we present an approach to evaluating the sequence and structure similarity of a very large number of hairpins with an application to the identification of pre-miRNA candidates. In a first step, we demonstrate that our vectorial representation of RNA structures and the Euclidian distance in the multidimensional space consequently defined is comparable with the sequence/structure similarities identified by LocARNA—a conventional structural clustering method. In a second step, we observe that known pre-miRNAs tend to populate a specific region of the multidimensional space defined by the principal components of a vectorial representation of all candidate structures. We therefore use the position of known precursors in this multidimensional space to identify the region of interest and select the candidates populating it.

### 2.1 Vectorial representation of sequence and structure

**Fig. 1.**
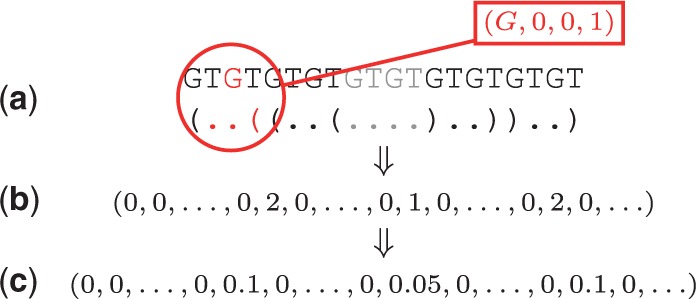
Example of a vectorial representation. (**a**) The characteristics of a single position are determined, which include the nucleotide and whether the previous, current and following positions in the secondary structure are unpaired (0), left/right paired (1/2), or located in the terminal loop (3). (**b**) Portions of the final vector illustrating the counts. Each vector position refers to a particular nucleotide type and the neighboring pairing status, from (A, 0, 0, 0) to (G, 3, 3, 3). (**c**) Portions of the normalized vector obtained from (b), each position is divided by a constant such that the sum of all components is 1

We use a vectorial representation for candidate precursors which summarizes key features of the primary/secondary structure of a given stem-loop. The representation we chose, after considering several options and selecting the one that best matched the results of conventional clustering (Supplementary Materials), consists of a vector of normalized counts. To build this vector, we use a sliding window of length 3 (a triplet) that scans the precursor candidate (Fig. 1). At each step, a position in the vector is incremented. The appropriate vector position is mapped considering whether each nucleotide within the window and with respect to the minimum free energy (MFE) structure is the left/right-hand side of a base-pair, an unpaired nucleotide on the stem, or part of the terminal loop, and, additionally, which base is present at the midpoint of the window. We have, thus, a vector with 256 positions. After scanning the entire precursor, each position in the vector is normalized by dividing its counts by the length of the sequence.

A similar representation has already been used to represent feature vectors of RNA stem-loops in the context of training a support vector machine (SVM) (Xue *et al.*, 2005) and was amongst the representations we have considered (Supplementary Materials). The representation we use here is richer than the one proposed by these authors in the sense that it distinguishes the situation where a given position is the left or right-hand side of a base-pair instead of simply being a paired position and it also represents unpaired nucleotides in the stem region or the terminal loop differently. In this way, information about asymmetrical loops and bulges in the stem is captured by the vector counts.

### 2.2 Conventional structural clustering

To identify clusters of high sequence-structure similarity, we apply a clustering procedure based on RNA sequence-structure alignment. For this purpose, we used LocARNA, which is one of the fastest and most accurate tools for multiple RNA sequence alignment (Will *et al.*, 2007). LocARNA performs Sankoff-style simultaneous alignment and folding (Sankoff, 1985). This approach generates high-quality alignments that take structural similarity into account. Notably, the structural information is not required *a priori* but can be inferred, in parallel to the alignment process, based on an RNA free energy model. LocARNA achieves its short run-times for pairwise alignment because it needs to consider only significant base pairs. The associated cluster pipeline generates a hierarchical cluster tree by applying an average-linkage clustering (unweighted pair group method with arithmetic mean—UPGMA) to the matrix of pairwise LocARNA distances. This pipeline was validated by a re-clustering of Rfam and could reproduce Rfam families with good precision at high average recall.

In the case of clustering miRNA candidates, we do not have any prior knowledge of clusters. Therefore, we need to define a reasonable partitioning of the cluster tree into an optimal number of clusters. For this purpose, we apply a variant of the Duda rule (Duda *et al.*, 2001) implemented in the tool RNAsoup (http://www.bioinf.uni-leipzig.de/∼kristin/Software/RNAsoup/). To this purpose, a subtree is reported as an optimal cluster if the sum-of-squared errors for two clusters is not significantly smaller than what is expected by chance (Kaczkowski *et al.*, 2009). The significance level can be controlled by *k*. The larger is the value of *k*, the larger the difference of squared error allowed before a subtree is split into two clusters. In our case, the error of a cluster is determined via the free energy of its consensus structure and the MFEs of its individual sequences. The MFE of single sequences is calculated by RNAfold (Hofacker *et al.*, 1994). The consensus structure and energy are calculated by RNAalifold (Hofacker *et al.*, 2002) based on a multiple LocARNA alignment of the subtree.

### 2.3 Principal component analysis of vectorial representations

The vectorial representation of the stem-loop structures used in this work captures information about sequence/structure features but, in general, the dimensions of these feature vectors are not independent. Furthermore, all vectors will always have zero values in some dimension as some combinations of left-/right-hand paired and unpaired nucleotides are not possible in actual RNA structures. To reduce the number of dimensions and to ensure we represent our structures in a space with independent dimensions, we readily eliminate dimensions with zero variance. In practice, for a sufficiently large dataset, this will only eliminate dimensions for which all vectors have a value of zero. Over the remaining dimensions, we perform a PCA thus obtaining a space of linearly independent coordinates. Additionally, each dimension of the vectorial representation is scaled to unit variance before performing the PCA.

### 2.4 Evaluation

#### 2.4.1 Randomization procedure

A randomization procedure is used to compare our approach to the results obtained for samples of the datasets using conventional structural clustering in terms of the proportion of correctly assigned cluster members. This procedure allows us to estimate the likelihood that our values were obtained by chance or as a result of the way our candidates are spatially distributed in the principal components space. To obtain the proportion of correctly assigned cluster members in the randomized version of the samples, we keep each candidate in the same position of the principal components space but we shuffle their identities, i.e. we randomly select two candidates and we swap their coordinates, repeating the process until all candidates have had their coordinates swapped. After having performed the random swapping of candidates, we calculate the centroids of each cluster in the shuffled space and the resulting proportion of correctly assigned cluster members. A similar procedure is used to obtain random samples of the median distance of known precursors to their centroid.

#### 2.4.2 Sampling the datasets for performance evaluation

To assess the performance of both our method and TripletSVM, four groups of samples of different sizes were prepared for each dataset. Each sample group was divided in training sets and testing sets, both with the same number of positive and negative examples. Each sample group is made of 1000 samples. The positive examples in the training set of each sample are a random subset of a pool of known miRNAs, with a single representative per miRNA family (5%, 10%, 20% or 50% of the pool) and the remaining pre-miRNAs in the pool make up the positive examples of the corresponding testing set. The negative examples in both the training and testing sets of each sample are random subsets of the candidates having the same size of the corresponding positive examples. Our method uses only the positive examples in the training set as a reference from which to compute the distance to the elements in the testing set, whereas TripletSVM, for each sample, is trained using both the positive and negative examples of the training set and is evaluated against the testing set.

## 3 DISCUSSION

### 3.1 Vector representation reflects structural clustering

To assess the adequacy of our approach with respect to its ability to identify regions of structural similarity in a way that resembles conventional sequence/structure clustering, we adopt the following procedure. We use LocARNA to perform hierarchical structural clustering over 100 samples of 1000 randomly chosen stem-loops drawn from the *D.melanogaster* and *A.gambiae* datasets, always including the entire set of known miRNAs for each organism, and we determine the optimal partition into clusters applying a tree node evaluation rule for various significance levels called *k*-levels (the details of this procedure are described in Section 2). For low values of *k*, the procedure produces clusters with highly similar structures. An increasing value of *k* allows for more dissimilar structures to be included in the same cluster, therefore producing a lower number of clusters with an increasing number of structures.

We then represent each structure from the samples using a vectorial representation summarizing sequence/structural features, in an effort to capture the key elements distinguishing the various hairpins. These feature vectors contain, however, both interdependent dimensions and dimensions with different variance. To obtain a linearly independent set of dimensions, we perform a principal components analysis (PCA) over the vectorial representations mapping them to their principal components representation (feature space).

To determine whether our representation of the candidates in the principal components space reflects the structural clusters found by LocARNA for the different *k* levels, we calculate the proportion of correct assignments, which measures the ratio of cluster members that are closer to their assigned cluster centroid as opposed to a centroid of another cluster. The cluster centroid is calculated by determining the average position of the cluster members each dimension at a time. The distribution of this measure in our 100 samples is then compared with its distribution in a randomized version of our spatial representation of the candidates, where candidate positions are kept but candidate identities are shuffled.

The comparison with the randomized version of the spatial distribution of candidates allows us to address the fact that the significance of the absolute distances of each candidate to its cluster centroid in the feature space can only be determined comparatively. For instance, some clusters may have only one member, in which case it will always coincide with its cluster centroid and, more generally, it may happen that variations in distance of a candidate to its assigned cluster centroid for different *k*-levels, or even different vectorial representations, may partially reflect the overall density of the candidates rather than a better evaluation of structural similarity.

Table 1 shows that, for both datasets, a large proportion of cluster members are found closer to their cluster centroid than to the centroid of any other cluster. For the most heterogeneous clusters that are obtained at *k*-level 0.90 the proportion of correctly assigned cluster members is about two-thirds, and it rises more than 80% for the structurally more homogeneous clusters obtained at *k*-level 0.00. The comparison with the randomized datasets shows that the results are statistically significant, i.e. these results are well above what one would hope to obtain by chance or simply due to the way candidates are spatially distributed.

**Table 1.** Evaluation of vectorial representations

*k*-level	*Anopheles gambiae*			*Drosophila melanogaster*		
	Correctly assigned (%)	*P* value	Average Cluster size	Correctly assigned (%)	*P* value	Average cluster size
0.00	83.60	8.58*e*−87	3.05	82.10	3.45*e*−125	3.29
0.10	82.50	1.69*e*−87	3.33	81.24	1.92*e*−120	3.54
0.20	81.21	8.68*e*−84	3.70	80.01	4.31*e*−113	3.89
0.30	79.30	2.37*e*−76	4.27	78.24	3.31*e*−108	4.44
0.40	77.09	9.22*e*−65	5.31	76.08	1.64*e*−96	5.44
0.50	74.12	2.24*e*−55	7.61	72.80	1.43*e*−84	7.54
0.60	71.23	2.76*e*−42	12.09	69.45	1.01*e*−61	11.44
0.70	68.70	1.05*e*−31	17.24	67.41	1.74*e*−54	15.32
0.80	68.14	6.01*e*−26	19.52	66.07	9.62*e*−44	17.77
0.90	67.37	2.57*e*−22	21.08	65.72	4.01*e*−37	20.09

*Note*: For each k-level, the table shows the percentage of correct assignments in the datasets of *A.gambiae* and *D.melanogaster*, the *P*-value of Welch’s two-sample *t* test comparing the observed correct assignments with a randomized version of each dataset shuffling the correspondence between candidates and their vectorial representation, and the average number of cluster members.

### 3.2 Known precursors are clustered together

Using the same samples from the datasets presented in the previous section, we can observe that despite the fact that not all known precursors are grouped together in the same cluster by LocARNA at any *k*-level (data not shown), they are however significantly close and restricted to a limited portion of the feature space. In fact, if we take the centroid of the known precursors and calculate the median distance of each known precursor to the centroid, we obtain a value which is much smaller than what would be expected by chance (*P*-value = 8.00 × 10^−^^60^ for *A.gambiae*, *P*-value = 8.94 × 10^−71^ for *D.melanogaster*, estimated using the randomization procedure described in Section 2.4.1).

### 3.3 Distance to known precursors is good predictor

The results shown in the previous section suggest that known precursors tend to concentrate in a particular region of the feature space. This region, however, is also densely populated by other precursor candidates. Because the region where known precursors are found is inserted in an area of great density, it cannot be identified by a purely unsupervised approach. Therefore, we take the coordinates of all known precursors and use them to identify the closest candidates. This method has the advantage of allowing for different pre-miRNA structural clusters to emerge around subsets of known precursors. The number of candidates that are included in the acceptance region is controlled by the maximum distance allowed to the closest pre-miRNA.

The larger the permitted distance, the greater the chance of selecting a region that includes all interesting candidates, but at the expense of enlarging the number of false positives. The Youden index (Youden, 1950), *J*, defined as *max_c_*{TPR (*c*) − FPR (*c*)}, i.e. the maximum difference between the true positive rate (TPR) and the false positive rate (FPR) over all cutoff values, *c*, is a standard method to select the best compromise in such a trade-off. The optimal cutoff value, c*, is the cutoff for which *J* = TPR (c^*^) − FPR (c^*^).

**Fig. 2.**
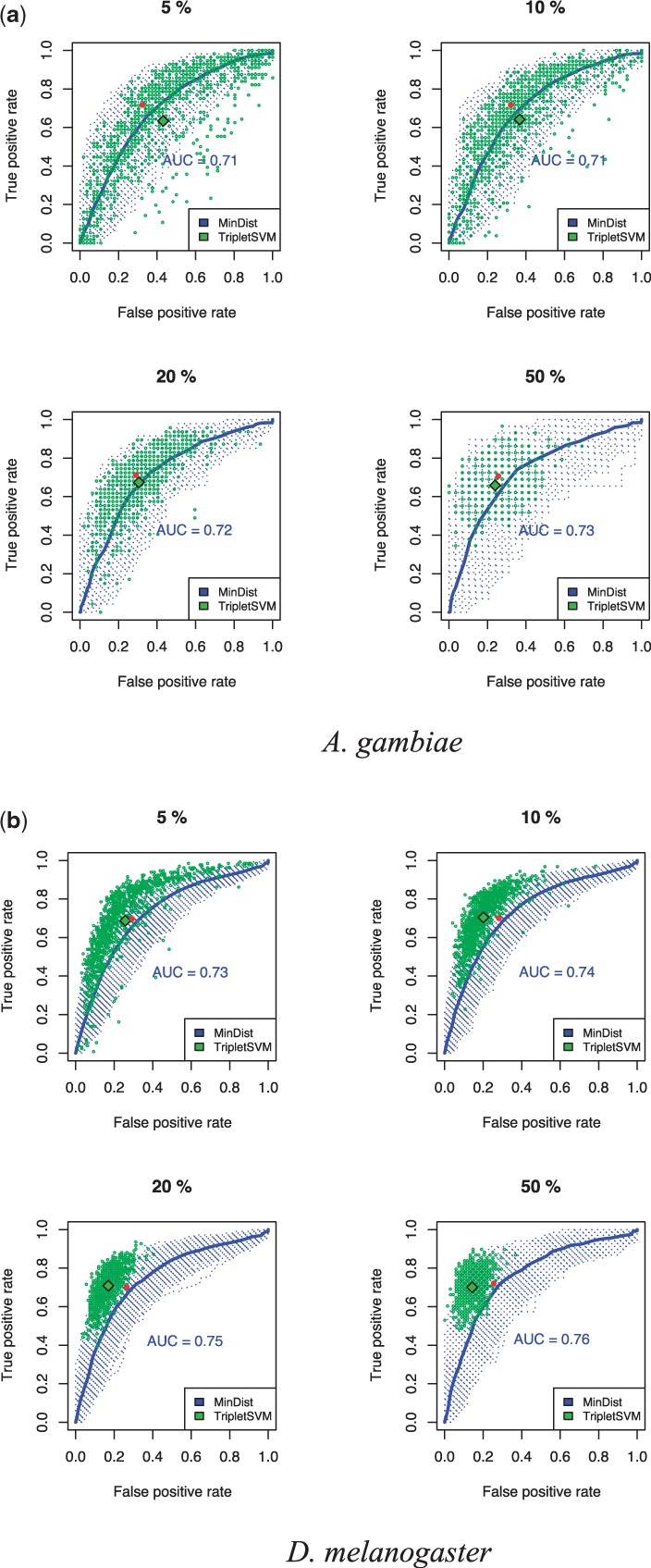
ROC curves for the minimum distance (MinDist) to pre-miRNAs method and the performance of TripletSVM over 4000 samples equally divided into four groups. Each group uses 5%, 10%, 20% or 50% of the known precursors of (**a**) *A.gambiae* and (**b**) *D.melanogaster* to set up the positive examples of the training set. The positive examples of the testing set are made up by the remaining precursors. In both sets, the negative examples are samples of the set of candidates. ROC curves for each individual sample are shown in dashed lines and the average curve across the range of cutoff values is shown in a solid line. The red dot represents the average performance of the MinDist method over all samples considering the optimal cutoff for each sample. The green dots represent the performance of TripletSVM on each sample, whereas the green diamond refers to its average performance

To estimate the optimal cutoff, we consider subsets of known precursors as reference and calculate the true/false positive rate with respect to the remaining known precursors and other candidates (see detailed description in Section 2). Figure 2 shows the receiver-operating characteristic (ROC) curves for *A.gambiae* and *D.melanogaster* when using samples of 5%, 10%, 20% and 50% of known precursors as reference and computing the trade-off between the true/false positive rates with respect to the remaining precursors and an equal number of sampled candidates. Each figure shows the ROC curves of 1000 such samples as well as the average curve, computed as the average performance over all samples across the full range of cutoff values. Additionally, the figures also show the average performance of our method, computed as the average TPR and FPR across all samples for the optimal cutoff on each sample (note that this may be significantly different from the optimal cutoff calculated on the average ROC curve).

The optimal cutoff in each of these ROC curves can be interpreted as the best choice of maximum distance allowed between a structure and the closest precursor so that the former may be included in the acceptance region. We have observed that there is a log-linear relation between the value of the average optimal cutoff and the percentage of known precursors that is used as reference (*R*^2^ = 0.998, for *A.gambiae*, and *R*^2^=0.989, for *D.melanogaster*). Because the best choice of cutoff cannot be directly determined for the entire set of known precursors, we estimate it by extrapolating the log-linear model. The estimated optimal cutoff can be interpreted as the best choice of maximum distance to include additional (yet unknown) precursors with the least number of false positives.

Using the estimated optimal cutoffs, the selected regions include 23.5% (77 366) and 23.5% (67 619) of all candidates from the *A.gambiae* and *D.melanogaster* datasets, respectively.

### 3.4 Comparison with other methods

TripletSVM (Xue *et al.*, 2005) is a classifier based on a SVM that purports to determine whether a given stem-loop is a pre-miRNA. The features considered by this SVM are quite similar to those of the TripletS vectorial representation that is described in the Supplementary Materials. It is also, to our knowledge, the only single-genome method whose source code is made available and which includes the necessary routines to re-train the model with new data. Many other single-genome methods exist (Mendes *et al.*, 2009), but their time complexity makes their use in the classification of hundreds of thousands of candidates unfeasible. TripletSVM was trained using positive examples from samples of known precursors and negative examples from samples from the candidate set (a detailed description is given in Section 2). A graphical representation of the performance of TripletSVM in each of the 4000 samples (evenly distributed between training sets using 5%, 10%, 20% and 50% of the annotated pre-miRNAs), as well as the average performance in each group of samples, is shown in Figure 2. Table 2 shows the sensitivity, specificity and the *F*_1_ measure for TripletSVM as well as our method across training sets including varying proportions (from 5% to 95%) of known precursors.

**Table 2.** Sensitivity (TPR), Specificity (1 − FPR) and the *F*_1_ measure 
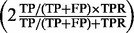
 of TripletSVM and MinDist computed as the average performance across all samples for training sets whose positive examples consist of a fraction of known pre-miRNAs in *Anopheles gambiae* and *Drosophila melanogaster*

% known	*A.gambiae*						*D.melanogaster*					
	MinDist			TripletSVM			MinDist			TripletSVM		
	Sensitivity	Specificity	*F*_1_	Sensitivity	Specificity	F_1_	Sensitivity	Specificity	*F*_1_	Sensitivity	Specificity	F_1_
5	0.72	0.68	0.71	0.63	0.57	0.61	0.70	0.71	0.70	0.69	0.74	0.71
10	0.72	0.68	0.71	0.64	0.73	0.67	0.70	0.72	0.71	0.70	0.80	0.74
20	0.71	0.71	0.71	0.67	0.69	0.68	0.70	0.73	0.71	0.71	0.83	0.76
50	0.71	0.74	0.72	0.66	0.76	0.69	0.72	0.75	0.73	0.70	0.86	0.76
80	0.70	0.80	0.74	0.64	0.78	0.69	0.75	0.75	0.75	0.69	0.86	0.75
90	0.74	0.82	0.77	0.63	0.78	0.68	0.77	0.78	0.77	0.68	0.87	0.75
95	0.83	0.79	0.81	0.64	0.78	0.69	0.79	0.81	0.80	0.68	0.87	0.75

The average performance of our method in *A.gambiae* is superior to that of TripletSVM, and slightly worse in *D.melanogaster*, except for sample groups containing a greater number of known precursors. The slightly better performance of TripletSVM in the *D.melanogaster* dataset is the result of a tendency for having comparatively higher specificity but similar sensitivity. This is probably due to the fact that MinDist is sensitive to the inclusion of heterochromatic sequences in this dataset, which introduces greater variability in terms of sequence/structure features. As a consequence, the variation between the features of pre-miRNAs and those of other stem-loops with more regular features becomes less apparent. It is worth noting, however, that despite achieving a slightly better average performance in *D.melanogaster* for sample groups containing a lower number of known precursors, the actual performance of TripletSVM in these samples varies greatly from one sample to the other, alternating between very good and very poor performances, particularly for the 5% sample group, as is shown in Figure 2(b), which is a major disadvantage when exploring recently sequenced genomes, for which few clear homologs with which to seed the search for new pre-miRNAs are generally available.

TripletSVM also bears the inconvenience of requiring negative examples which are inevitably chosen under the assumption—however plausible and defensible—that miRNA precursors are rare with respect to the overall number of candidates, but one cannot generally guarantee that hairpins which would normally be processed by the miRNA-maturation pathway are not being included in the negative training set. Our approach, despite assuming all candidates to be false positives for the purposes of performance evaluation, does not use this information to shape the acceptance region and, since it does not try to identify the optimal margin between positive and negative examples, it is also less likely to suffer from overtraining. Additionally, by reflecting sequence/structure similarity in a way comparable with conventional structural clustering, our method offers a better interpretation of the decision rule that is made when selecting candidates.

### 3.5 Transcriptional potential assessment further restricts the number of candidates

The number of candidates obtained after our structural analysis (77 366 for *A.gambiae*, and 67 619 for *D.melanogaster*), albeit considerably lower than the original candidate set (328 829 for *A.gambiae* and 287 469 for *D.melanogaster*), is still quite numerous. A plausible interpretation of these results is that, despite their structural similarity to known precursors, the majority of these candidates are not pre-miRNAs due to other factors. Chiefly among these is the fact that most remaining candidates are probably not efficiently transcribed or are playing different biological roles. This illustrates the often ignored distinction between having an adequate secondary structure and actually being transcribed and processed.

A straightforward way to address the need to assess the transcriptional potential of the candidates is the observation that many fall within regions that have been annotated. Genomic locations with no annotation or which have been annotated as introns may contain miRNA precursors, but candidates that overlap regions annotated as exons, transposons or other non-coding RNAs are less likely to contain pre-miRNAs. If we filter out non-viable candidates by this criterion, our candidate set is reduced to 44 210 for *A.gambiae* and 40 582 for *D.melanogaster*.

If we additionally restrict our search to putative miRNA cluster members, which are very common genomic organizations of miRNA precursors in metazoans, we can lower the number of candidates by considering only those which are found in the vicinity of known pre-miRNAs. The price to pay for this reduction is that we risk missing yet unidentified miRNAs that happen to occur in genomic locations far from previously identified precursors, or which are not part of a miRNA cluster. By selecting candidates with viable annotation and at a genomic distance not greater than 50 kb [as suggested in Baskerville and Bartel (2005)] from pre-miRNAs, we reduce our candidate set to 439 for *A.gambiae* and 1604 for *D.melanogaster*.

After having determined, for each dataset, the reduced list of candidates, a more detailed analysis was performed for two different approaches, both using LocARNA as a method to perform structural hierarchical clustering of our candidates along with the annotated precursors. Unlike before, we do not use a parameterized partition rule to enumerate our clusters at different *k*-levels. Instead, we use criteria aimed at identifying miRNA genomic clusters. The first approach consists in identifying, starting from the leaves of the similarity tree, the smallest structural clusters that include at least one known precursor, and within these clusters we enumerate all subsets of stem-loops that are located in close genomic proximity to each of the precursors in the structural cluster. This way we can identify the candidates which are both structurally similar and occurring close to each given precursor in the genome. The second approach drops the requirement that a known precursor be present and simply identifies leaves in the LocARNA similarity tree, extracting the top-scoring clusters in terms of SCI (Washietl *et al.*, 2005) (Structure Conservation Index). Additionally, we enumerate all subsets of stem-loops that are in close genomic proximity to each other, regardless of whether a known pre-miRNA is present. This way we try to identify candidates which are both similar and in the vicinity of one another. Subsets of stem-loops identified in the second approach which happen to be included in the output of the first approach are discarded.

**Fig. 3.**
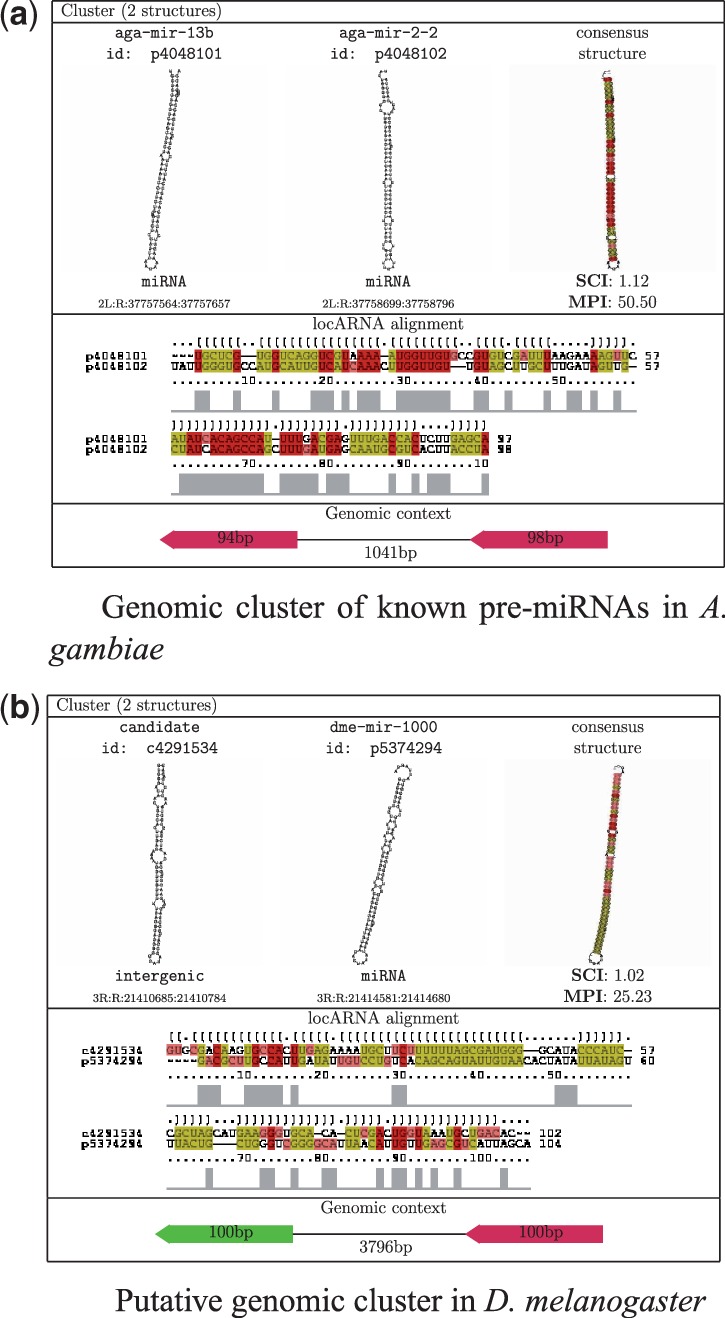
Genomic clusters of pre-miRNAs. Shown are the secondary structure of both stem-loops, the consensus structure along with the SCI (structure conservation index) and the MPI (mean pairwise identity), the LocARNA alignment and a representation of their genomic loci

The first approach identifies 108 and 422 candidates in the extracted clusters from *A.gambiae* and *D.melanogaster*, respectively, of which 5 and 11 were in the genomic vicinity of the precursors included in their respective clusters. A total of 9 and 23 potential genomic clusters of pre-miRNAs (not to be confused with structural clusters) corresponding to the relevant subsets of stem-loops of each structural cluster were identified using this approach for *A.gambiae* and *D.melanogaster*. Respectively, 4 and 13 were composed entirely of known pre-miRNAs at a median distance of 3327 and 237.5 bp while 5 and 10 contained precursor candidates at a median distance of 12 424 and 22 820 bp (see Supplementary Materials for the list of most promising cluster candidates). The genomic clusters exclusively made up of precursors, as the one shown in Figure 3(a), attest the ability of our method to identify structurally homogenous pre-miRNA genomic clusters while the clusters which include new candidates, as seen in Figure 3(b), may indicate new instances of this type of genomic organization and plausible miRNA precursors.

The second approach, which purports to identify potential genomic clusters where all members are non-annotated is, naturally, limited to those candidates which happen to be included in the initial set and are therefore close to known precursors in the genome, but which are structurally more similar to each other than to any pre-miRNA. This approach identified 481 and 1618 candidates in the extracted structural clusters for *A.gambiae* and *D.melanogaster*, respectively, of which 81 and 147 were not more than 50 kb away from another stem-loop in the same structural cluster. The potential genomic clusters identified for this approach total 7 and 65 at a median distance of 17 880 and 12 797 bp, respectively for *A.gambiae* and *D.melanogaster*. Interestingly, there are several instances of identical or highly similar candidates in both datasets that make up genomic clusters. Some of these clusters (Supplementary Materials) have up to three identical candidate members and are evenly spaced across the genome. They are either the result of a duplication event (more specifically, triplication) or they correspond to instances of long repeats, transposons or other repeat sequences. In any case, the fact that they were selected based on their structural similarity to known pre-miRNAs hints at the closeness of these structures and supports the much discussed possibility that repetitive sequences may serve as a dormant repository of stem-loops which could be co-opted as pre-miRNAs (Smalheiser and Torvik, 2005).

## 4 CONCLUSION

We have presented a method to assess the sequence/structure similarity of a large dataset of hairpins in search for novel pre-miRNAs, and we have placed these candidates in a multidimensional space in a way that reflects their structural characteristics. The portion of the multidimensional space selected around the known pre-miRNAs purports to include most structures which have the potential of being efficiently recognized by the cellular miRNA-processing machinery.

The fact that this region is very dense in terms of the number of precursor candidates it contains, tells us that a large number of genome locations have the potential to generate stable and robust structures which present sequence/structure similarities to known pre-miRNAs. The use of annotation information helps reducing the number of selected candidates but after this filtering step, which is nevertheless dependent on the quality and breadth of the available annotation data, they remain in the tens of thousands. Therefore, there is either an exceedingly large number of pre-miRNAs in these datasets or, more plausibly, most of these candidates are not efficiently transcribed but could otherwise be recognized as miRNA precursors.

The initial set of candidates extracted from the genomes of *A.gambiae* and *D.melanogaster* and described in Mendes *et al.* (2010) consisted of 2 245 014 and 1 316 305 candidates, respectively. The total number of candidates, after applying stability and robustness measures described in earlier work and the structural analysis along with the annotation filtering detailed in this article, is reduced by two orders of magnitude to 44 210 and 40 582.

Unlike many machine learning approaches to the identification of miRNA precursors that use features of the sequence and secondary structure to provide a classifier, our approach does not need to postulate a set of negative examples. In fact, we contend that if the purpose is to characterize the structures which have the potential of being recognized by the enzymes involved in miRNA maturation, one needs to reduce one’s dependence on the positive set as well, because it will most likely not be representative—it suffices to observe that the set of recognizable structures is surely larger than the set of all the pre-miRNAs contained in the genome and that these two sets are subject to different evolutionary constraints. In our work, information about known precursors is used merely to pinpoint a region of interest in our multidimensional representation of sequence/structure features. Admittedly, this approach is not guaranteed to identify the entire portion of the feature space where structures recognizable by the miRNA-processing machinery are located, because such a subspace is surely much larger than the examples that could ever be instantiated in any genome. However, our method outperforms a machine learning approach based on a SVM in one dataset and has comparable performance with the other, for larger training sets. For sample groups with a greater number of positive examples it outperforms the machine learning method in both datasets.

One can further limit the candidates to those occurring in the genomic vicinity of known precursors and which could, therefore, be part of miRNA clusters together with pre-documented pre-miRNAs. This approach produces a greatly reduced set of candidates (439 for *A.gambiae*, and 1604 for *D.melanogaster*), even using a very liberal definition of miRNA cluster. This significant reduction of the number of candidates, albeit at the expense of the ability to identify novel miRNAs located elsewhere in the genome, elicits both the possibility of experimental validation and further detailed computational analyses. To this effect, it was possible to identify several plausible miRNA clusters with structurally similar stem-loops by performing conventional structural clustering over this reduced set, along with an analysis of their genomic disposition.

*Funding:* FCT—Fundação para a Ciência e a Tecnologia (INESC-ID multiannual funding) through the PIDDAC Program funds, by the PTDC program [PTDC/AGR-GPL/098179/2008] and under project [PEst-OE/EEI/LA0021/2011] (to N.M. and A.T.F.), the Agence Nationale de la Recherche [BRASERO project BLAN06-3 138859, MIRI project BLAN08-1 335497] (to N.M. and M.F.S.) as well as by the European Research Council under the European Community’s Seventh Framework Programme (FP7/2007-2013) / ERC grant agreement no [247073]10 held by M.F.S., the German Research Foundation [DFG grants BA 2168/3-1 to S.H. and R.B., BA 2168/4-1 to R.B.], the Excellence Initiative of the German Federal and State Governments [grant EXC 294 to R.B.].

*Conflict of Interest*: none declared.

## Supplementary Material

Supplementary Data
