# Supplementary material for: Navigating the unexplored seascape of pre-miRNA candidates in single-genome approaches
Source: Bioinformatics. 2012 Oct 10;28(23):3034–41. doi: 10.1093/bioinformatics/bts574 (PMC3516144; doi:10.1093/bioinformatics/bts574)
Supplement: Supplementary Data [file supp_28_23_3034__index.html]

Navigating the unexplored seascape of pre-miRNA candidates in single-genome approaches — Navigating the unexplored seascape of pre-miRNA candidates in single-genome approaches — Supplementary Data 

# Navigating the unexplored seascape of pre-miRNA candidates in single-genome approaches

## Supplementary Data

files

**Files in this Data Supplement:**

- Supplementary Data - tex file
